# Chicken muscle mitochondrial content appears co-ordinately regulated and is associated with performance phenotypes

**DOI:** 10.1242/bio.022772

**Published:** 2016-12-01

**Authors:** Antonio Reverter, Ron Okimoto, Robyn Sapp, Walter G. Bottje, Rachel Hawken, Nicholas J. Hudson

**Affiliations:** 1 Agriculture and Food, Commonwealth Science and Industrial Research Organisation, Brisbane, Queensland 4067, Australia; 2 Cobb-Vantress Inc., US-412, Siloam Springs, AR 72761, USA; 3 Department of Poultry Science, University of Arkansas, Fayetteville, AR, USA; 4 School of Agriculture and Food Science, University of Queensland, Building 8117A, Gatton, Queensland 4343, Australia

**Keywords:** Mitochondria, Muscle, Growth, Development

## Abstract

Mitochondrial content is a fundamental cellular bioenergetic phenotype. Previous work has hypothesised possible links between variation in muscle mitochondrial content and animal performance. However, no population screens have been performed in any production species. Here, we have designed a high throughput molecular approach to estimate mitochondrial content in commercial broilers. Technical validity was established using several approaches, including its performance in monoclonal DF-1 cells, cross-tissue comparisons in tissues with differing metabolic demands (white fat<breast muscle<drumstick muscle<heart muscle) and, as a negative control, a near absence of mtDNA amplification from whole blood. We screened breast muscle and thigh muscle in 80 birds individually phenotyped for 11 growth and development traits. Substantial individual variation (fivefold) was discovered in both breast and thigh muscle mitochondrial content. Interestingly, across birds we detected a very strong positive relationship between breast and thigh content (correlation coefficient 0.61; *P*<0.0001), consistent with coordinate regulatory control across the musculature. Further, breast muscle mitochondrial content is negatively correlated with breast muscle yield (−0.27; *P*=0.037), abdominal fat content (−0.31; *P*=0.017) and carcass yield (−0.26; *P*=0.045). Therefore, low breast muscle mitochondrial content is associated with more muscular birds possessing higher abdominal fat, the latter being in line with biomedical models of obesity. Finally, thigh mitochondrial content is negatively correlated with the bow out leg defect (−0.30; *P*=0.011). Overall, our data point to mitochondrial content as a promising consideration in predictive modelling of production traits.

## Abstract (precis)

**Summary:** We have detected variation in muscle mitochondrial content across chickens. This variation relates to phenotypes and also indicates coordinate regulation of mitochondrial biogenesis across the musculature.

## INTRODUCTION

The poultry industry is currently improving its flocks for commercial traits relating to growth and development, while attempting to minimise the appearance of structural defects. This is currently achieved through a combination of genetic selection, nutrition and other management practices. Genetic selection occurred historically through direct selection on desirable phenotypes. In the post-genomics era it has exploited single nucleotide polymorphism (SNP) genotyping to enable DNA marker-assisted selection practices (Lahav et al., 2006). The combination of the two practices has transformed domestic chickens over the last century to the modern animals with their precocious growth rates, dramatically increased muscle mass and markedly improved feed efficiencies (Paxton et al., 2010; Siegel, 2014).

In principle, the DNA-based selection approach can mitigate the need to measure expensive phenotypes such as feed efficiency, or make predictions about individuals that do not directly express the phenotype, such as the contribution of male genetics to female reproductive characteristics. However, predicting phenotype from genotype is challenging even for industries that have an animal resource with a small effective population size and high levels of inbreeding. In chickens, the current accuracy of genomic prediction for a typical complex trait of moderate heritability is 54% (Lourenco et al., 2015). This is strong enough for implementation in a breeding strategy but there would be value in further improvements. One possible avenue is to develop biomarkers complementary to DNA sequence information. If these are practical and economical, they could be implemented in parallel to genetic testing and the two sources of information integrated for stronger predictions. Alternatively, any SNP subsequently found to be associated to the new biomarker could help refine the existing genomic predictions and therefore be delivered through the current DNA prediction pipeline.

In this study we describe the implementation of a high-throughput method to screen chickens for the cellular phenotype of mitochondrial content, and then to connect any detectable variation to performance characteristics that have industrial and biological relevance. We chose mitochondrial content as a potential biomarker because it is a highly fundamental bioenergetic phenotype governing aerobic capacity (Holloszy, 1975), with implications for numerous metabolic conditions. For example, given that mitochondrial content relates to both muscle structure and metabolism, we previously hypothesised it to be relevant to both the physiology of the live animal (Hudson, 2009) and also meat quality via an effect on post-mortem metabolism (Hudson, 2012). We know that across species there is enormous variation in tissue mitochondrial content. For example, the very athletic hummingbird possesses a pectoralis mitochondrial content of 35% (Mathieu-Costello et al., 1992), whereas the much more sedentary broiler has a ∼tenfold smaller value of 4% (Kiessling, 1977). The former has great athleticism, but also very high food demand and becomes torpid each night to fend off the threat of starvation; however, modern broilers are sedentary and among the most feed-efficient vertebrates known.

The profound modification in skeletal muscle from a red mitochondrial-rich aerobic ancestral state to a white, mitochondrial-poor glycolytic derived state observed in all growth and efficiency selected breeds of numerous production species [e.g. Large White and Yorkshire pigs (Jing et al., 2015; Sales and Kotrba, 2013; Vincent et al., 2015), callipyge sheep (Jackson et al., 1997a,b), *MSTN* mutant cattle (Kambadur et al., 1997; McPherron and Lee, 1997) and sheep (Clop et al., 2006)] further implicates the mitochondrion as a likely player. In fact, these observations imply that a gradual diminishment in mitochondrial content is a general feature of the domestication of hyper-muscular, feed-efficient groups. However, it has not been clearly established how much population variation in tissue mitochondrial content exists in any production species, or if it does exist which phenotypes it would best inform. Here, we associated variation in tissue mitochondrial content across 80 birds to 11 commercial phenotypes that were largely uncorrelated with each other, allowing us to explore various facets of growth, metabolism and development in some detail.

## RESULTS

### Sybr green assays

**Fig. 1.**
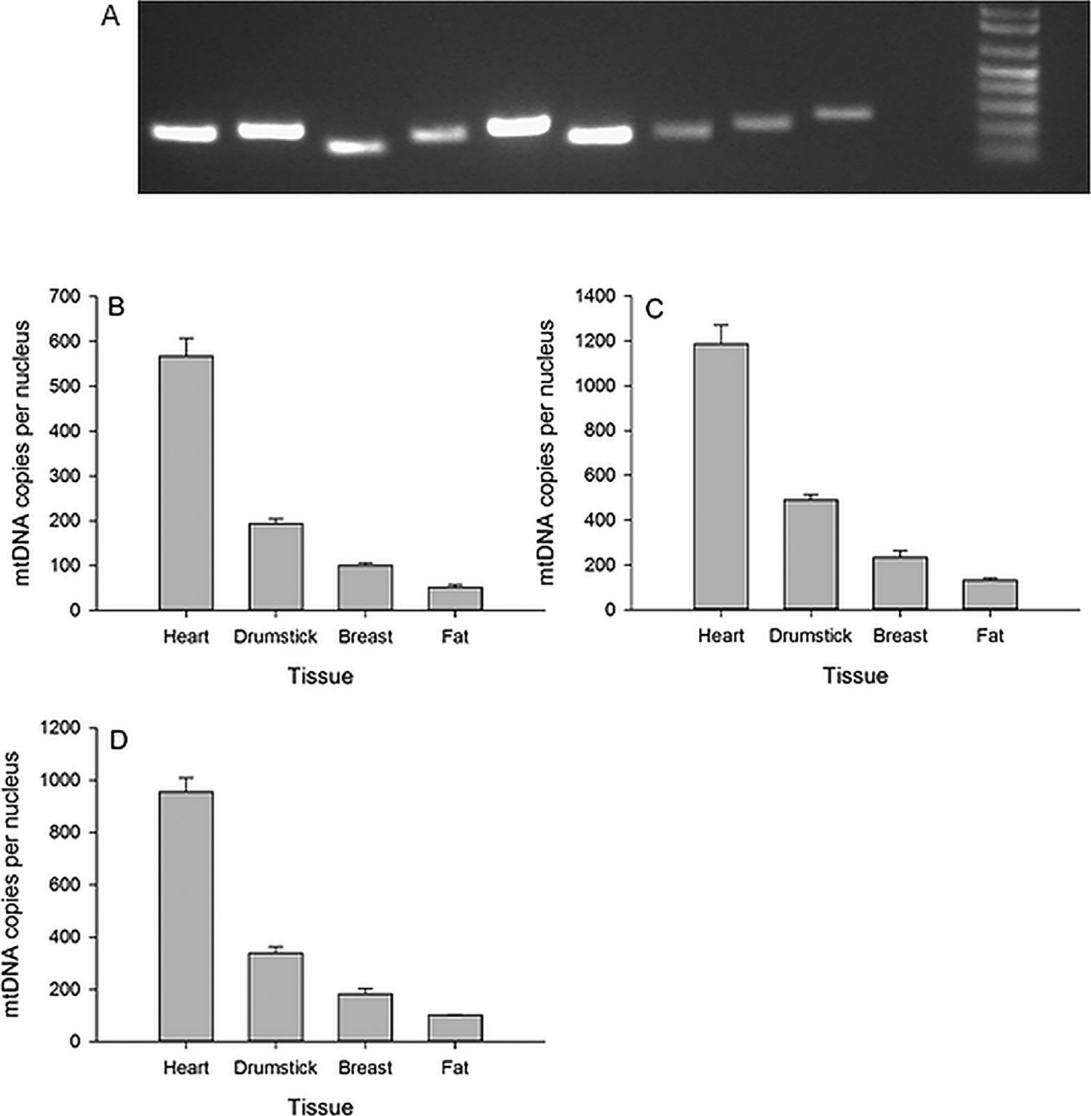
**The performance of the SYBR Green-based primer pairs.** (A) The amplicons produced by the 9 Sybr Green primer pairs after 30 min of gel electrophoresis at 70 V. The order is as follows: *ND2*, *ND3*, *ND4L*, *ND6*, *COX1*, *COX3*, *ATP1A1*, *GDF8*, *GAS7* plus 1Kb ladder, with predicted amplicons sizes of 200, 210, 150, 199, 227, 171, 170, 200 and 246 bp respectively. The discriminatory ability of *ND2* (B), *ND3* (C) and *COX1* (D) corrected against *GDF8* to estimate cross tissue mitochondrial content. All results based on SYBR Green qPCR chemistry. Results are presented as mean±s.d.

Good quality DNA was purified from all four tissues. The A_260/280_, A_260/230_, and DNA concentrations (ng/µl) were 2.01, 1.99, 78.5 (heart); 2.01, 1.67, 67.2 (drumstick); 1.99, 2.14, 83.0 (breast) and 2.00, 1.86 and 67.0 (fat). Following conventional PCR, a single amplicon of the predicted size was identified by gel electrophoresis in all cases (Fig. 1A). Further exploration by qPCR identified a small shoulder on the *ND6* and *ATP1A* dissociation curves with the remaining assays yielding unique peaks. PCR efficiencies ranged from 85–109% with six assays >90%. Overall, all assay combinations were convincingly able to discriminate the four tissues in the expected order of mitochondrial content, i.e. heart>drumstick>breast>fat. We next explored the various combinations in more detail looking for technical reasons to select one assay over another.

In a single plate containing the appropriate comparison assays, *ND4L* and *ND6* showed double the mtDNA copies of *ND3*, *COX1* and *COX3*. This applied when corrected against both *GAS7* and *ATP1A1* nDNA. One explanation is possible pseudogenic amplification. That is, the *ND4L* and *ND6* primers may have amplified an unidentified Numt in addition to the bona fide mitoDNA segment. Consequently, we rejected the *ND4L* and *ND6* primers. The *ATP1A1* primers possessed a small shoulder on the dissociation curve so were rejected. The *GDF8* nDNA primers were preferred as they were designed with a coding exon and therefore considered less likely to differ for the comparisons between individual birds. After several rounds of exploration we found *ND2*, *ND3* and *COX1* corrected against *GDF8* to perform consistently well (Fig. 1B). Of these three, *ND2:GDF8* showed the largest overall discrimination in terms of fold change between the tissues (Table S2). *ND2* also predicted lower overall mtDNA copy values than *ND3* and *COX1*, perhaps indicating greater mtDNA reaction specificity.

Focussing on the preferred *ND2:GDF8* qPCR assay we computed an mtDNA copy number of 99 for nuclei in breast muscle. According to Kiessling (1977) this should equate to a mitochondrial content of ∼4%. Using our estimated fold changes across tissues we predicted chicken fat (51 mt copies per nuclei) to have a mitochondrial content of 2.1%, chicken drumstick (193 mt copies) to have a mitochondrial content of 7.8% and chicken heart (567 copies) to have a mitochondrial content of 22.7%.

### Taqman duplex mitochondrial content assay

The next sections describe the performance of the Taqman duplex assay across a range of empirical circumstances, including tissue sampling site, across tissue comparisons and performance in DF-1 monoclonal cells *in vitro*.

### Serial sampling

We explored the impact of tissue sampling depth on mitochondrial content in several birds. While we detected sources of variation, we did not uncover any systematic relationships. For example, the upper portion of the breast muscle yielded a higher content estimation than the mid and lower portions for bird 31018 but not bird 31017 (Fig. S1A,B). Next, a more thorough serial sampling methodology was applied to bird 31008, taking eight successive samples at increasing depth (Fig. S1C). The absolute values for bird 31008's estimation of mitochondrial copy number are higher than the other breast values we calculated (often >400). However, these serial samples were RNase treated and the DNA yield and purity were reduced in all cases. A recent paper gives a possible explanation for this phenomenon (Donà and Houseley, 2014). Focusing on the relative differences along the serial sampling, it is clear that there is likely some biological variation and care will be needed selecting the same tissue site for different birds and value in taking an average of more than one site per bird.

### RNase treatment

Across a number of different experiments we detected no systematic effect of RNase treatment on the output of the assay (Table S3). Given RNase treatment costs time and money and reduces DNA yield and purity, we elected to exclude RNase treatment during DNA extraction for our main population screen, in line with previous recommendations (Guo et al., 2009).

### DF1 cell culture

**Fig. 2.**
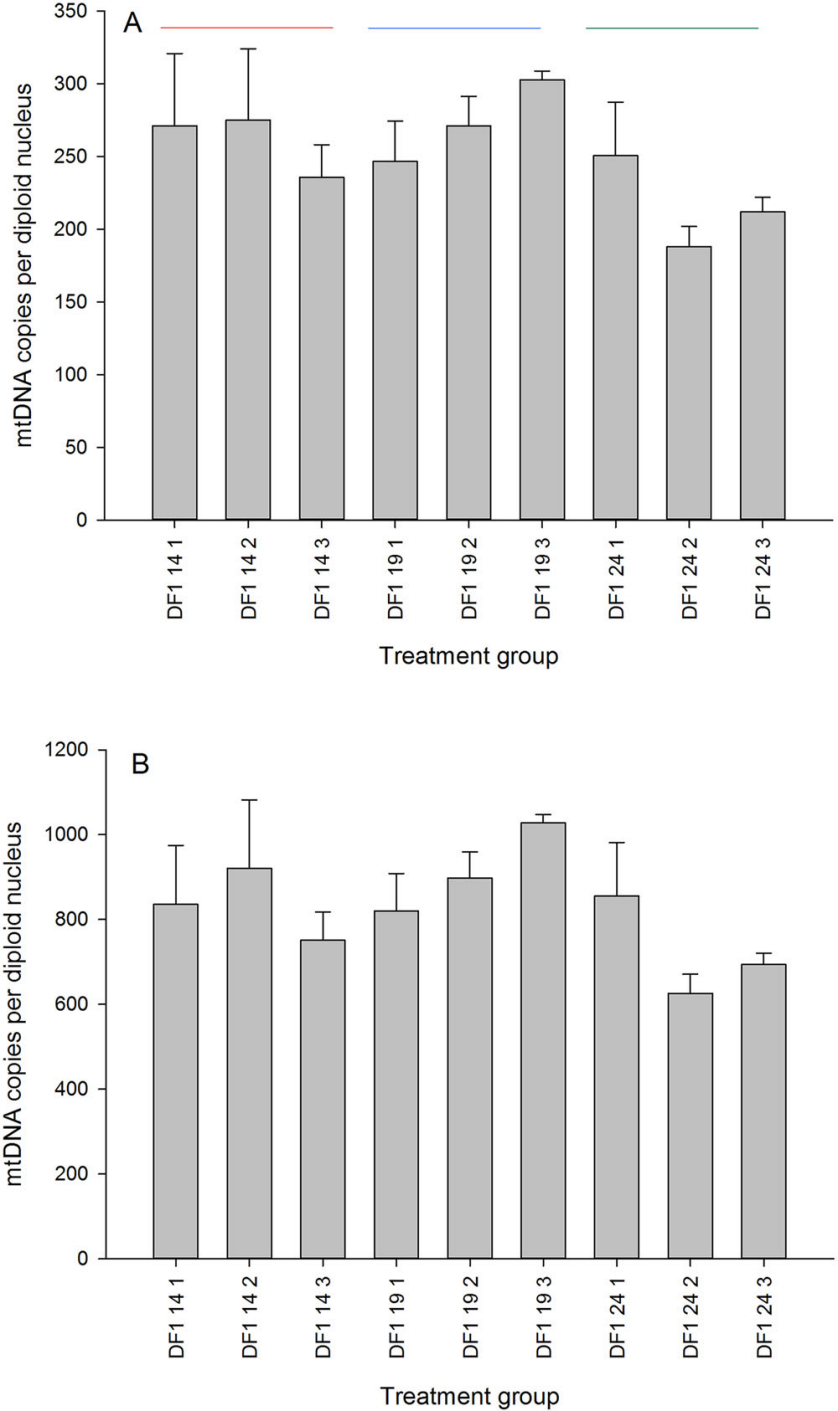
**The performance of the Taqman assay on monoclonal DF-1 cells grown to confluence.** There are three passages (14, 19 and 24 denoted by the coloured lines) and three biological replicates per passage (denoted as 1, 2 and 3). Data shown as means±s.d.

The averages for passage 1, 2 and 3 respectively are 260.7, 274.2 and 216.9 (Fig. 2). Some of this variation may be real biological variation (effect of cell culture conditions, cell confluence and other factors on cell metabolism) and some is technical variation due to DNA extraction effects. To better discriminate we explored the relationship between DNA yield and mtDNA copy number estimation for these samples, finding a negative correlation of −0.72 (Table S4). In the context of DF1 cell pellet extractions, higher DNA yields tend to produce lower estimates of mtDNA copy number. This relationship further reinforces the importance of running any tissue DNA extractions in parallel with similar amounts of starting material with an aim to generate consistent yields and purity. However, in the final analysis we found that working from data that has the differential PCR efficiency of the two reactions accounted for removes the statistical dependency on DNA yield.

### Tissue comparisons

**Fig. 3.**
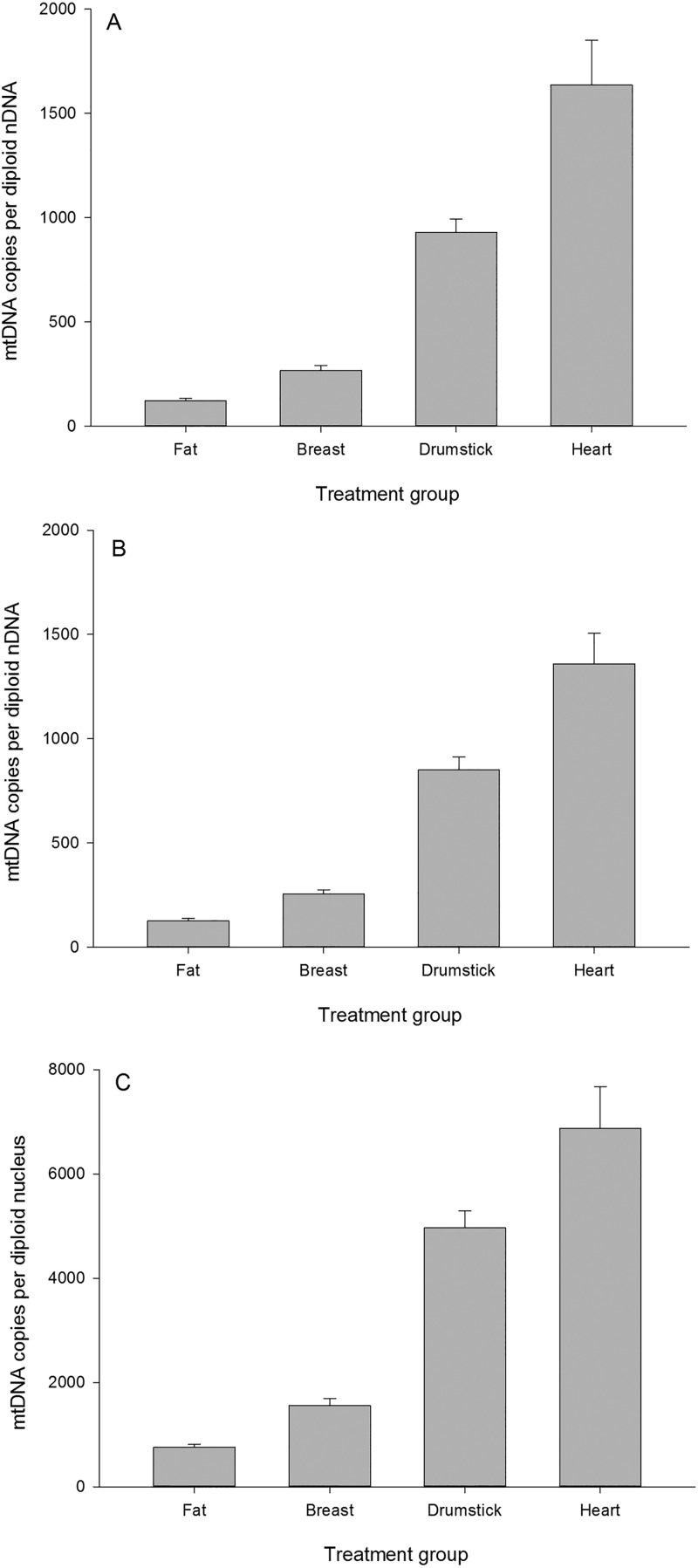
**Estimation of mitochondrial content across four chicken tissues using the Taqman assay.** The same samples were assayed independently on two independent runs (panel A and B). In panel C differential PCR efficiency has been accounted for using the same raw data as shown in panel A. Data shown as means±s.d.

Following the previous approach taken with SYBR Green we ‘ground-truthed’ the performance of the *ND2 GDF8* Taqman assay by comparing the following tissues in ascending order of mitochondrial content: white fat<breast muscle<drumstick muscle<heart muscle (Fig. 3A,B).

In line with the previous findings based on SYBR Green, the Taqman assay clearly discriminate the four tissues in the expected manner. The *ND2* (mtDNA) reaction has a PCR efficiency of 83% whereas the *MSTN* (nDNA) assay has a PCR efficiency of 96.5%. Accounting for the differential PCR provides the following estimates (Fig. 3C) for the four tissues. The relative differences in mtDNA copy number are largely unaltered, but the absolute estimates are considerably higher.

### Screening 80 commercial broilers for breast, thigh and blood using the Taqman assay

The three breast values (A, B and C) were indistinguishable from each other (Table 1), so we pooled them to produce a single averaged breast value per bird. We analysed the blood data separately from the main statistical model because the blood values are several orders of magnitude smaller than the muscle values. We found that 75% of the variation in mitochondrial content was explained by our statistical model (DNA yield, tissue, bird). Interestingly, uncorrected values (assumed 100% efficiency for both nDNA and mtDNA PCR reactions) are negatively affected by DNA yield, but the correction for differential PCR efficiency removes this dependency. This is an appealing outcome from a technical perspective as it means when we report corrected values we do not need to be as concerned about individual variation in DNA yield.

**Table 1.**
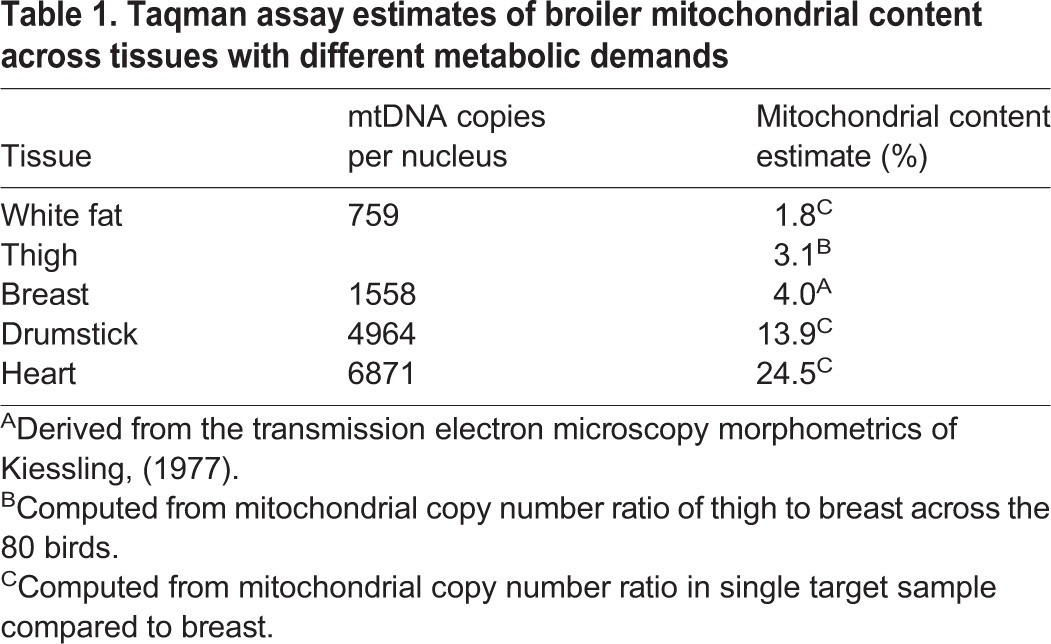
**Taqman assay estimates of broiler mitochondrial content across tissues with different metabolic demands**

The thigh values (Z) were lower than the breast values (A-C) (Table 2). This is contrary to our biological expectation as the thigh muscle, equivalent to the human upper leg quadriceps, is presumably subject to more mechanical work than the breast muscle in a largely flightless bird. It is also contrary to our findings previously made on the drumstick which is equivalent to the human lower leg (i.e. calf) muscle. We noticed that the thigh DNA extractions themselves appeared to be systematically different to the breast ones, with substantially lower yields (average ∼50 ng/μl, as opposed to ∼75 ng/μl). To further explore the effect of yield, we analysed the mitochondrial content estimates for only those birds for whom the thigh yield was at least as high as the breast – these 21 birds still had lower estimates of mitochondrial content in thigh than breast (data not shown) implying that the result is probably not a consequence of the DNA extraction method.

**Table 2.**
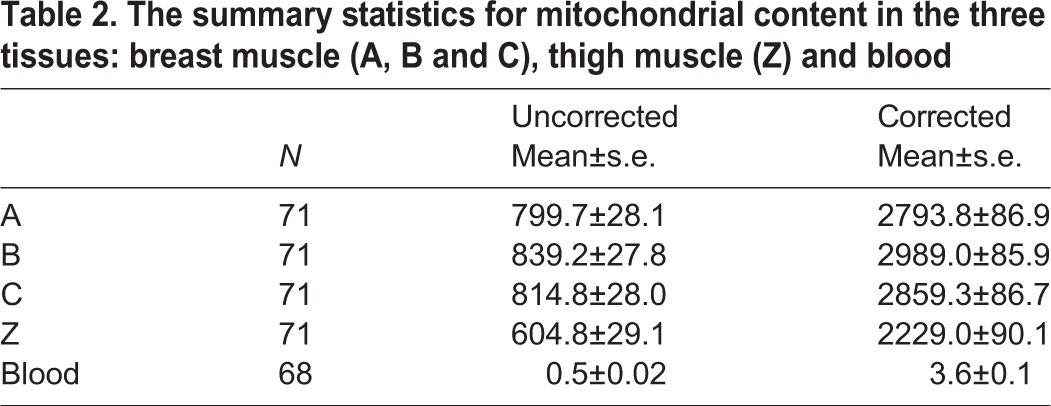
**The summary statistics for mitochondrial content in the three tissues: breast muscle (A, B and C), thigh muscle (Z) and blood**

**Fig. 4.**
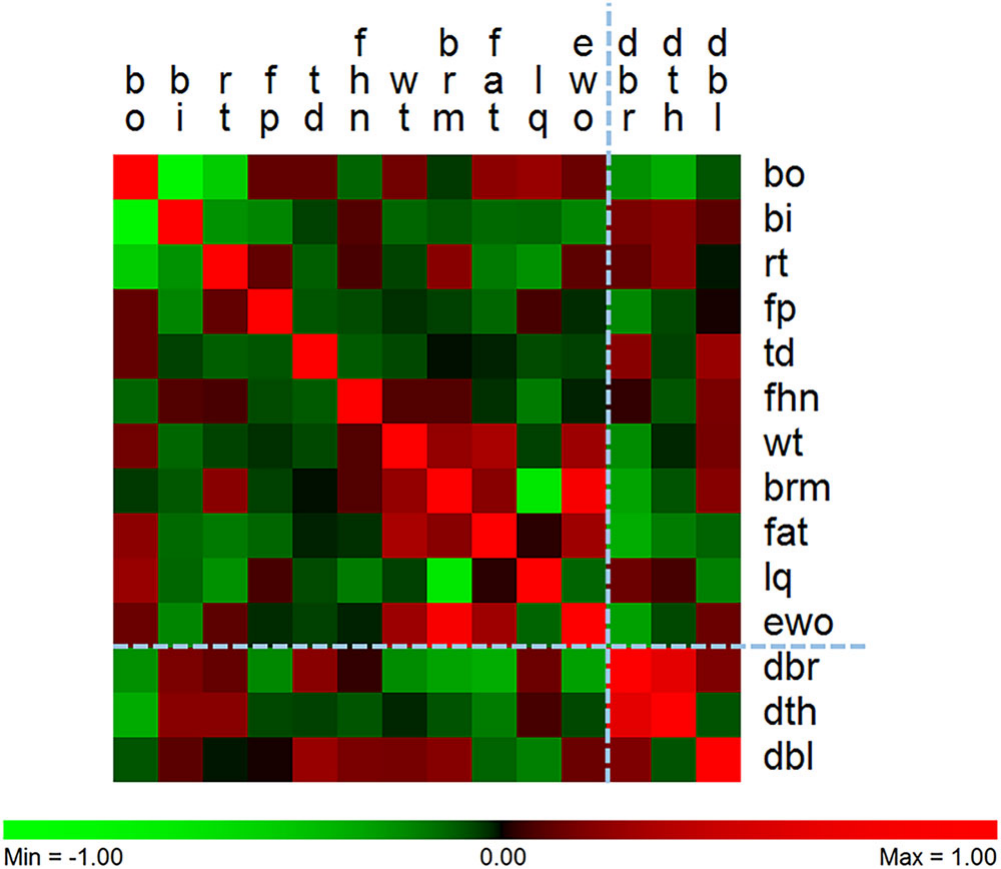
**The relationship between phenotypes and tissue-specific mitochondrial content based on patterns of co-correlation.** There are similar patterns of correlation across the bird phenotypes. Phenotypes are in columns and tissue-specific mitochondrial content are in rows. DBR, DTH and DBL refer to breast, thigh and blood mitochondrial content respectively. The two skeletal muscles are more related to each other than the blood. The phenotypes cluster such that breast meat and carcass yield cluster with body weight, and two leg defects (BI and RT) cluster together. Breast muscle mitochondrial content is negatively correlated with breast muscle yield (−0.27; *P*=0.037), abdominal fat content (−0.31; *P*=0.017) and carcass yield (−0.26; *P*=0.045). Thigh mitochondrial content is negatively correlated with the bow out leg defect (−0.30; *P*=0.011).

The blood mtDNA estimates were extremely low but detectable in all samples. Next we performed correlation analyses to associate the individual corrected values for the three mitochondrial content estimates (breast, thigh and blood) to each other and the various phenotypes (Table S5; Fig. 4). All correlation values and effect sizes can be found in Table S5. This data matrix was used for hierarchical clustering on rows and columns (Fig. 4) to derive relationships among both phenotypes and mitochondrial content parameters. The clustering on rows is more robust than the clustering on columns in line with the number of cells driving the clustering relationships. This correlation output was also used to guide the decision as to which relationships we would explore more deeply, using least square means analyses for discrete phenotypes and regression analyses for continuous phenotypes.

**Fig. 5.**
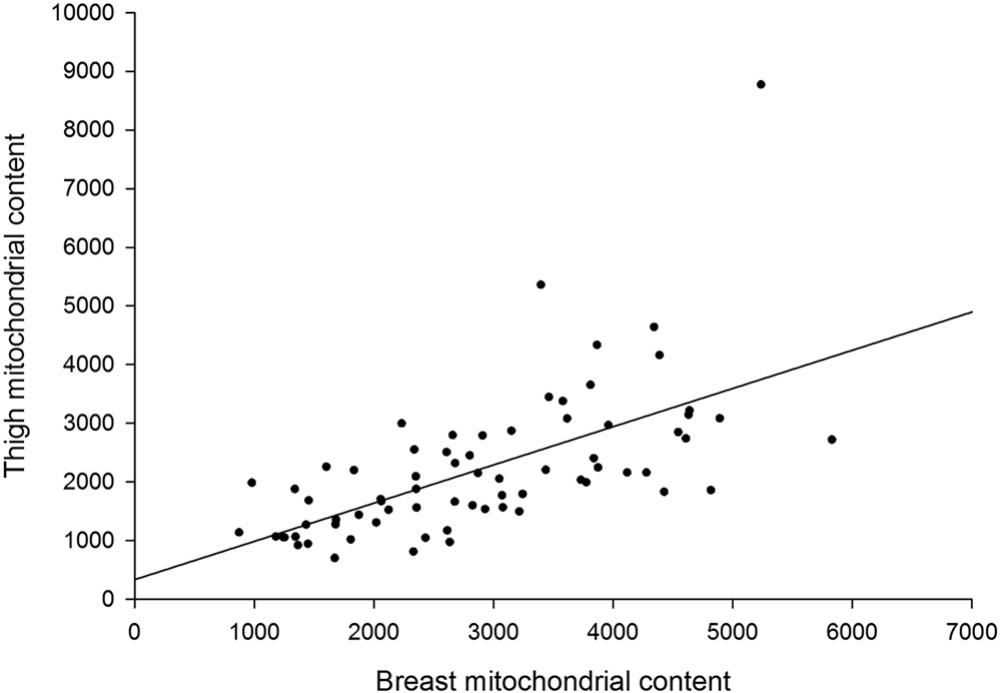
**The strong positive relationship between the mitochondrial content of breast muscle and thigh muscle.** Birds with high mitochondrial content in one muscle tissue also tend to have high content in the other muscle tissue (Pearson correlation coefficient 0.61; *P*<0.0001). The spread across the population in terms of both breast and thigh muscle mitochondrial content fold change is substantial (∼fivefold).

The 11 phenotypes provided are an excellent resource in the sense that, with the exception of three of the leg defects [bow out (bo), bow in (bi) and rotated thigh (rt)] they are largely unrelated to each other. A major new finding is the very significant relationship between breast mitochondrial content and thigh mitochondrial content (correlation coefficient 0.61; *P*<0.0001; Fig. 5). That is, birds with relatively high breast content also possess relative high thigh content. Breast mitochondrial content correlates with breast meat yield (−0.27; *P*=0.037), abdominal fat (−0.31; *P*=0.017), estimated carcass yield (−0.26; *P*=0.045) and thigh mitochondrial content (0.61; *P*=0.001). Thigh mitochondrial content correlates with bowed out leg (−0.30; *P*=0.01). That is, birds with low mitochondrial content thigh muscle are more likely to have bowed out leg (Fig. S2). We also noted a graded (but non-significant) response of femur head necrosis severity and successive reductions in thigh mitochondrial content (Fig. S3). Blood mtDNA content was not significantly associated with any phenotype, although there was a trend towards significance for tibial dysplasia (0.23; *P*=0.074).

### Phenotypes

The summary statistics for the 11 phenotypes are shown in Table 3.

**Table 3.**
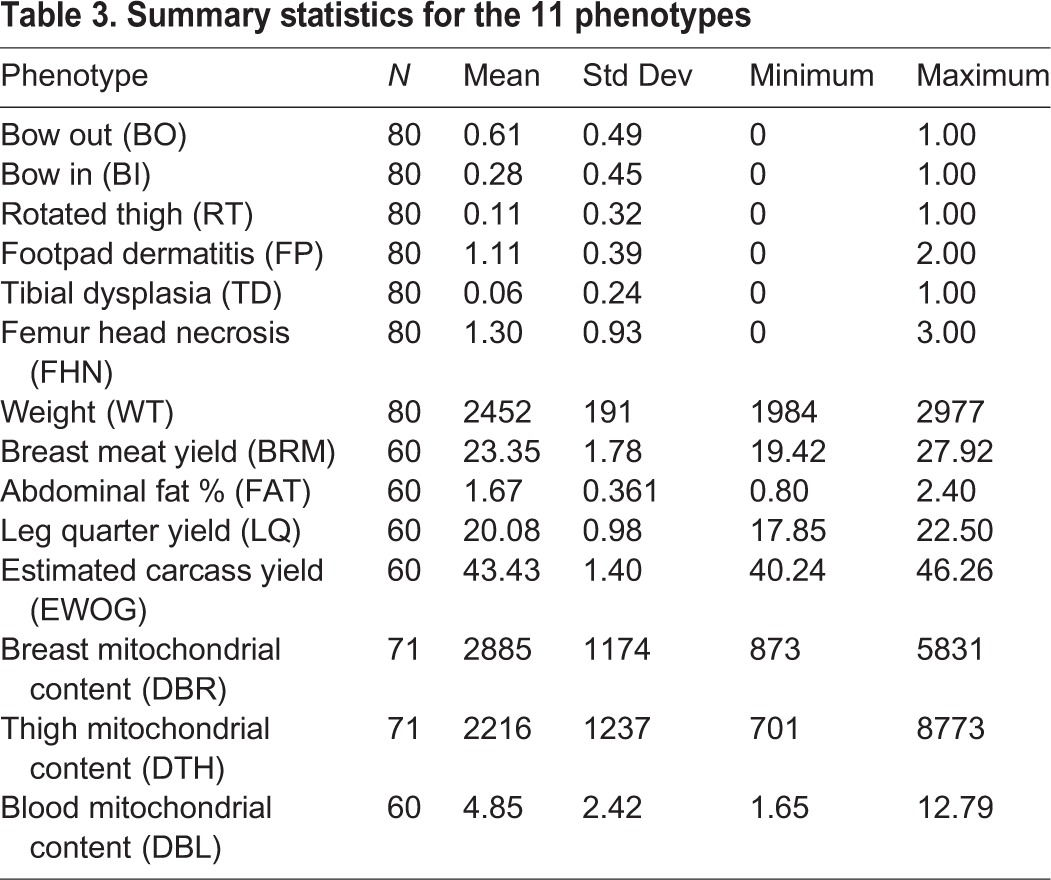
**Summary statistics for the 11 phenotypes**

## DISCUSSION

We have developed a sensitive, repeatable high throughput molecular assay to detect variation in mitochondrial content in chickens. Despite the inbred nature of the chicken populations being investigated and the homogenous fibre composition of the breast muscle, we have identified substantial individual to individual variation in both breast and thigh muscle, detected relationships with several commercially and biologically important phenotypes and present a first compelling line of evidence consistent with systemic control of skeletal muscle oxidative capacity in birds.

Application of the assay to blood DNA produced very low yields, implying our primers avoided amplification of any pseudogenic mtDNA that may be present in the nuclear genome. Unlike mammalian red blood cells which contain no mitochondria, low levels of mitochondria are known to be present in avian erythrocytes (Stier et al., 2013). We explored technical repeatability in *in vitro* circumstances using monoclonal DF-1 chicken cells and found reasonable consistency between passages and biological replicates. We detected some within-tissue variability through serial sampling methods which emphasised the importance of tissue site during sample collection. As proof of concept we ran the assay across chicken tissues expected to vary in mitochondrial content (white fat<breast<drumstick<heart), finding our molecular data broadly met those expectations. In line with previous authors, we found that RNase A treatment strongly interfered with DNA yield (Donà and Houseley, 2014) so we elected to omit it from our final DNA extraction protocol.

We detected substantial population variation in both pectoralis and thigh muscle mitochondrial content, in the order of fivefold. Given sources of error could accumulate during tissue excision, DNA extraction and subsequent pipetting presumably some of this variation will be technical, with the remainder representing true biological variation. The mean mitochondrial content in pectoralis was estimated at 2880 mtDNA copies/nDNA copies. If we assume this equates to a content of 4% (Kiessling, 1977) then we can predict chicken white fat, thigh, drumstick and cardiac muscle to have contents of 1.8%, 3.1%, 13.9% and 24.6%, respectively. Based on comparative mitochondrial content data across mammals, heart possesses a mitochondrial content between 22% and 37% (Barth et al., 1992; Larsen et al., 2012); and based on human subjects, leg muscle ranges from 4-15% (Larsen et al*.,* 2012). White fat would be expected to be very low, but we could not find published values. While we could not find exact figures for chicken for any other tissue than breast muscle, our drumstick and cardiac estimates seem reasonable given those values previously reported in mammals.

Most strikingly, we detected a highly significant positive relationship between thigh and pectoralis mitochondrial content (correlation coefficient 0.61; *P*<0.0001). That is, an individual bird with relatively high pectoralis content will also possess relatively high thigh content. This result strongly implies coordinate regulation of mitochondrial content across the skeletal musculature. Our outcome appears consistent with basic physiological expectation. For example, endurance training has a systemic, rather than local, effect on the musculo-skeletal system (Benzi et al., 1975). The extreme whole animal performance seen in highly athletic species, such as pronghorn antelope (Lindstedt et al., 1991) and hummingbirds (Mathieu-Costello et al., 1992), is seen as a complex trait integrating numerous components of the musculature, not just a single muscle. Finally, recent work has identified circulating master regulators of mitochondrial content, such as irisin (Cunha, 2012; Kelly, 2012), that are thought to act systemically across multiple muscle tissues.

We are not aware of any precedent for our result in the literature, perhaps because population screens of mitochondrial content across multiple tissues are rare or even absent. Bai et al. (2004) performed a tissue mitochondrial content screen in humans, but they screened only one muscle and the only phenotypes under investigation were suspected mitochondrial disorders. This means Bai et al. (2004) had no scope to identify the across-tissue relationship we detected and also could not explore relationships to non-pathological complex traits. Similarly, Curran et al. (2007) performed a large population screen comprising 1259 Mexican Americans and 1088 Caucasians for mitochondrial content, but they based their analysis entirely on blood samples. Because of this, their study had at least two limitations from our perspective: firstly, they could not explore cross tissue relationships and secondly, the physiological relevance of mitochondrial content is not clear for blood in the way it is for the skeletal musculature.

In terms of relationship to the 11 phenotypes, we found breast muscle mitochondrial content to be negatively correlated with breast muscle yield (−0.27; *P*=0.037), abdominal fat content (−0.31; *P*=0.017) and carcass yield (−0.26; *P*=0.045). Therefore, low breast muscle mitochondrial content is associated with more muscular birds possessing higher abdominal fat. This looks to be very much in line with biomedical models of fat metabolism, for example Tanner et al. (2002) who found less oxidative muscle predisposes humans to obesity. Further, thigh mitochondrial content is negatively correlated with the bow out leg defect (−0.30; *P*=0.011). The specific connection of breast muscle mitochondrial content to breast-related phenotypes and thigh muscle mitochondrial content to a leg-related phenotype is noteworthy. Overall, our data point to mitochondrial content as a promising new consideration in agricultural biomarker development and predictive modelling of growth and developmental traits.

### Conclusions

This is the first time tissue mitochondrial content has been assayed across a population of production animals. Despite the inbred nature of the chicken populations being investigated and the homogenous fibre composition of the breast muscle, we have identified substantial individual to individual variation in both breast and thigh muscle, detected relationships with several commercially and biologically important phenotypes, and present a first compelling line of evidence consistent with systemic control of skeletal muscle oxidative capacity in birds.

## MATERIALS AND METHODS

### Mitochondrial content assay design

Mitochondrial content is typically measured directly through morphometric quantitation of serial transmission electron microscopy sections (Hoppeler et al., 1981) or by proxy using one or more of (1) lipid quantitation of cardiolipin, (2) biochemical measurement of citrate synthase enzyme activity, or (3) qPCR quantitation of mtDNA copies expressed relative to nDNA. We elected to proceed with the last of these given the very high sensitivity of qPCR, the fact it can be developed into a high throughput assay in a 384-well plate format, and because the poultry industry is now routinely taking DNA samples as part of its genetic marker-assisted selection strategy; the final assay also made use of Taqman primer/probe technologies run in duplex. However, prior to this we explored several mtDNA and nDNA combinations using the cheaper SYBR Green technology in single plex assay format in an effort to focus in on the most promising primer pairs. The next section outlines the approach we took in proceeding from a set of plausible primer design options to the one ultimately deemed most acceptable.

### SYBR Green qPCR assay

For mtDNA primer design, we aimed to design qPCR primers that would uniquely amplify a segment of haploid mtDNA whose abundance could be corrected against a unique segment of diploid nDNA. In most tissues comprising mononucleate cells this approach represents a correction for cell number. In a post-mitotic multinucleate tissue, like skeletal muscle, this approach is equivalent to a tissue volume correction, on the grounds that every nucleus services a given volume of cytoplasm. In all cases Primer3 software (http://simgene.com/Primer3) was used to design primers, accepting the default settings (150-250 bp amplicon size, 18-27 bp primer length, 57-63°C annealing temperature and control of GC content between 20 and 80%). We used a previously published method (Pereira and Baker, 2004) to identify and test those mtDNA genes for which no nuclear pseudogenes (so-called Numts) have been previously identified in chicken. Any primers that inadvertently amplified Numts in addition to the target mtDNA would compromise the assay. Based on the assessment of (Pereira and Baker, 2004) we prioritized 6 of the 13 mitochondrially-encoded genes for deeper exploration: *ND2*, *ND3*, *ND4L*, *ND6*, *COX1* and *COX3*.

Further, mtDNA is highly variable between and even within individuals (heteroplasmy), given its physical proximity to reactive oxygen species (ROS) production and lack of histone protection. The presence of high DNA variation could threaten primer binding fidelity. Consequently, it was decided to focus exclusively on mtDNA coding sequences on the grounds that these sequences are less likely to harbor SNP. mtDNA possesses no introns, so the entire coding sequence (start codon to stop codon) was identified in NCBI nucleotide (https://www.ncbi.nlm.nih.gov/nucleotide) in each case and pasted into Primer3. We downloaded the mitochondrial sequences from the following link which contains the entire *Gallus gallus* mitochondrial genome: http://www.ncbi.nlm.nih.gov/nuccore/nc_001323.1. The six coding sequences were also BLASTED against the latest assembly of the chicken genome to check for more recently discovered Numts. None were identified.

For nDNA primer design, we decided to focus on single copy genes to simplify the correction to nuclear content (two copies of the nDNA gene represents one diploid nucleus). Three nuclear genes were selected: *GDF8* (*Chromosome 7*), *ATP1A1* (*Chromosome 1*) and *GAS7* (*Chromosome 18*). The first two were selected because of their fundamental role in physiology and muscle development, and the third because it was found to sit in a region of very low genetic diversity in Cobb chickens (A.R., R.O., R.S. and R.H., unpublished data). *GDF8* was designed within the 373 bp comprising exon 1 of the gene. *GAS7* and *ATP1A1* primers were not forced within an exon. The primer sequences and basic bioinformatic information are summarized in Table S1. The relative performance of these qPCR assays was assessed on four chicken tissues where we had a strong expectation they would possess differing mitochondrial contents (white fat, breast, drumstick and heart). These were purchased from the refrigerated section of a butcher store in Brisbane, Australia (P & L Fresh Meats).

### DNA extraction

The four tissues (heart, drumstick, breast and fat) were independently minced using a scalpel blade at room temperature and overnight digested in proteinase K at 56°C. The following morning total DNA was extracted using the Qiagen DNeasy tissue ‘on column’ system following the manufacturer's instructions. Following the procedures outlined by Guo et al. (2009), no RNase treatment was applied. For this initial part of the analysis, it was assumed any carryover mRNA would be destroyed by the overnight incubation at 56°C given RNA lability and the absence of RNA stabilization chemistry. DNA was eluted in 200 µl RNase- and DNase-free water and spectrophotometry used to quantify abundance and purity. All four samples yielded a similar high quality DNA preparation. The four DNA samples were also visualized on a 1.8% ethidium bromide-stained agarose gel. An aliquot of each neat DNA sample was serially diluted tenfold to produce the following dilutions: 1:10, 1:100 and 1:1000.

### Polymerase chain reaction

A conventional 60°C PCR was run using Invitrogen's platinum Taq DNA polymerase with 1:10 breast DNA as template. The PCR products were visualized by 1.8% gel electrophoresis stained with ethidium bromide. Next, qPCR assays were run using the SYBR Green-based system on an Applied Biosystems ViiA7 sequence detection machine running 5 μl reactions in triplicate based on 1:10 diluted DNA, as previously described (Hudson et al., 2006). Each primer pair had a ‘no template’ control. All primers were also run on tenfold serially diluted samples for the purpose of calculating standard curves and PCR efficiencies. The downstream analyses were performed using ViiA7 software. Technical replicate dispersion, no template controls, standard curves and dissociation curves were all manually scrutinized as part of a basic quality control procedure, in addition to the automatic quality control performed by the software itself.

The mtDNA copy number was expressed relative to nDNA copy number, herein referred to as M2N for mitochondrial to nuclear content, using the following equation:

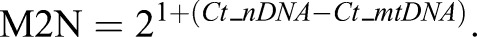


This equation converts the two Ct values (mtDNA and nDNA) into a mtDNA:nDNA copy number ratio, accounting for the fact the mitochondrial genome is haploid. For the purposes of these exploratory assays we did not account for PCR efficiency. With this single plex approach technical variation is a concern as the correction for nuclear DNA is based on an additional (independent) pipetting event. Our solution was to redesign our preferred assay (ND2 and GDF8) using a Taqman duplex approach. With this approach, both target mtDNA and normalization nDNA gene reactions take place in a single well, and the use of a probe in addition to the forward and reverse primers provides greater amplification specificity.

### Taqman duplex assay

**Fig. 6.**
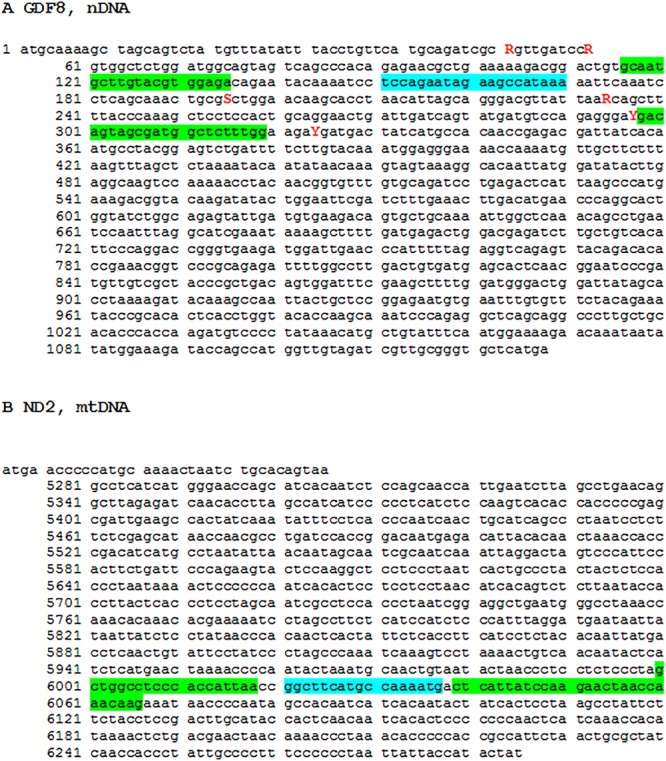
**The Taqman primer (green) and probe (blue) assays for nDNA *MSTN* and mtDNA *ND2*.** (A) nDNA MSTN, (B) mtDNA ND2. Red letters denotes SNP known to be segregating in Cobb birds. The *MSTN* and *ND2* nucleotide sequences were derived from accession numbers NM_001001461.1 and KF93904 (CDS 5247-6285), respectively.

The Taqman assay amplifying mtDNA (*ND2*) relative to nDNA (*GDF8*) was designed in collaboration with an Applied Biosystems technician (Fig. 6). In house AB software was used to optimize GC content, predict annealing temperature and estimate other parameters of interest. The *ND2* forward primer, reverse primer and probe had Tm of 59.5, 58.7 and 69.0 and GC contents of 58%, 36% and 40%, respectively. The *GDF8* forward primer, reverse primer and probe had Tm of 58.9, 59.9 and 68.0 and GC contents of 48%, 55% and 35%, respectively. Finally, we had to modify the *ND2* and *GDF8* primers (compared to the previous SYBR Green primers) to accommodate not only the position of the probe, but also to avoid binding to SNP known to be segregating in Cobb birds.

In line with Applied Biosystems technical recommendations, the VIC dye was tagged to the probe amplifying the more abundant target (mtDNA) and the FAM dye to the probe amplifying the less abundant target (nDNA). The more abundant target (mtDNA) was also setup as a ‘primer limited’ assay (3 μM each primer and 5 μM probe, compared to 18 μM each primer and 5 μM probe). This ensures that the more abundant target does not deplete the reaction ingredients before the less abundant target crosses the amplification threshold. 10 μl was chosen as a compromise volume reaction mix, small enough to be cost effective but large enough to reduce the relative impact of pipetting errors.

### Serial sampling of breast muscle and RNase treatment

We explored the effect of breast tissue depth by taking eight successively deeper samples providing a range from superficial to deep. We also explored the effect of + or – RNase treatment on the same samples (based on parallel samples ‘A’ and ‘B’ taken from bird 31018).

### DF-1 cell culture

In order to further explore the technical repeatability of the DNA extraction methodology, we grew a monoclonal immortal chicken embryo fibroblast (DF-1) cell line to confluence over three passages (14, 19 and 24) and with three biological replicates per passage, under standard cell culture conditions. With this approach an assumption was made that the mitochondrial content of these cells will be nearly identical, and therefore any differences detected can be likely attributed to technical error accumulated during either DNA extraction and/or implementation of the assay.

### Screening the breast, thigh and blood of 80 commercial broilers using the Taqman assay

We screened 80 industrial birds for three independent breast samples (A, B and C), a thigh sample (Z) and a whole blood sample. The samples were derived from animals from a single Cobb-Vantress broiler line. Male broilers were raised under standard industry practices and euthanized using carbon dioxide asphyxiation. Cobb-Vantress Inc. adheres to internal industrial standard guidelines for animal welfare and has standard operating procedures for euthanasia, dissection and other experimental techniques.

**Fig. 7.**
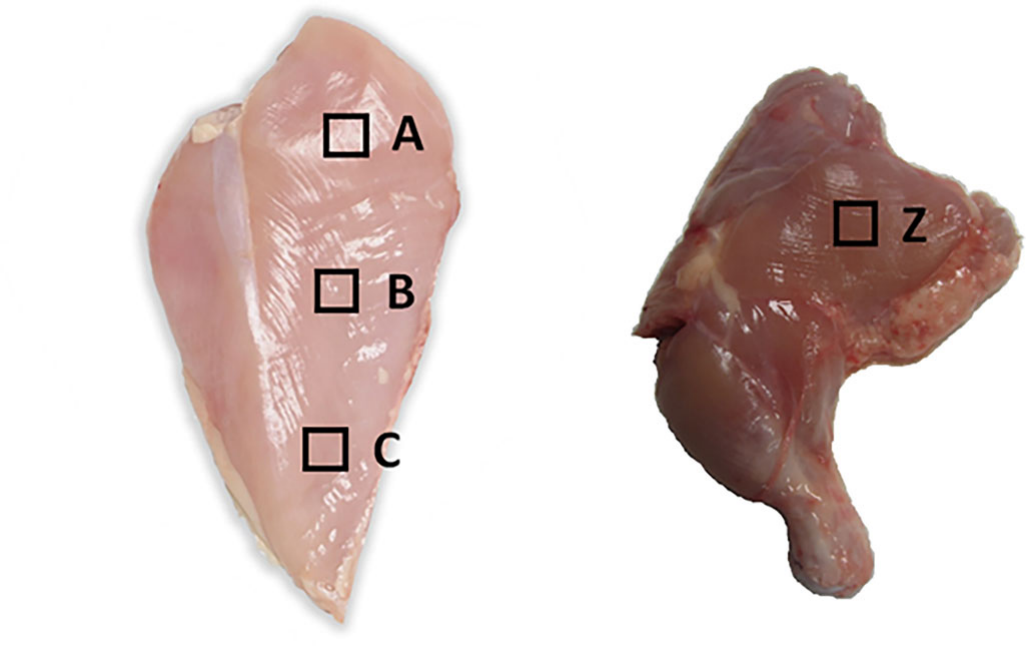
**The square boxes labelled on the pectoralis major (A,B,C) and iliotibialis lateralis (Z) indicate where tissue samples were taken for DNA extraction.** Except when otherwise specified, tissue was taken from just under the surface.

Muscle samples were taken from birds at 48 days of age. Three breast muscle samples of the pectoralis major were taken from each animal (A, B and C). All breast samples were taken down the centre axis of the pectoralis major. The A sample was taken from the thickest cranial region of the breast muscle, the C sample was taken from around 4 cm above the tip of the lower breast, and the B sample was taken from the middle of the iliotibialis lateralis, the large broad muscle that covers the outer thigh (Fig. 7). When multiple samples were taken for DNA isolation they came from deeper into the muscle from the same location. The samples were frozen on dry ice for transport and kept at −20°C until DNA isolation. All muscle samples were taken from around 2 mm below the outer membrane covering the muscle. The outer membrane and around 2 mm of muscle tissue was sliced off the sample before the muscle sample for DNA isolation was taken.

### Phenotypes

Eleven phenotypes were quantified at the time of assessment as follows: bird weight (WEIGHT) in ounces, score for legs bowed out [BOWOUT (BO), 0=unaffected, 1=affected], score for legs bowed in [BOWIN (BI), 0=unaffected, 1=affected], score for legs rotated [ROTATED LEG (RT), 0=unaffected, 1=affected], score for footpad dermatitis (FP, 0=unaffected; 1=mild; 2=moderate; 3=severe), score for tibial dysplasia (TD) at dissection (0=unaffected, 1=affected), breast meat yield (BRM) expressed as a percentage of carcass, abdominal fat percent (FAT) expressed as a percentage of carcass, leg quarter yield (LQ) expressed as a percentage of carcass, estimated carcass yield (EWOG) and score of femur head necrosis (FHN) at dissection [where the score is the sum of both legs (0=unaffected; 1=mild; 2=moderate; 3=severe)].

### Statistical analysis

A correlation analysis was performed to explore the relationship between mitochondrial to nuclear (M2N) DNA content across tissues and with the 11 recorded phenotypes. For categorical phenotypes (e.g. BO, BI, RT, FP, TD and FHN) this relationship was further characterized by fitting a one-way ANOVA model with the M2N at each tissue as the response variable, and a *t*-test performed on the least-square means of M2N estimated at each level of the categorical phenotype. Similarly, for continuous phenotypes (e.g. BRM, FAT, LQY and EWO), this relationship was further characterized by fitting a regression model with the phenotype and the M2N as explanatory variables, respectively. The procedures CORR, GLM and REG of the SAS 9.4 statistical software (SAS Institute Inc., Cary, NC, USA) were used. In order to protect against Type-I false positive rate we focused our analysis and discussion on pre-planned comparisons, namely the relationships between muscle mitochondrial content and the various phenotypes. The numerous relationships among the phenotypes themselves were largely ignored for the purposes of this study.
